# Topical Application of Dexamethasone‐Loaded Core‐Multishell Nanocarriers Against Oral Mucosal Inflammation.

**DOI:** 10.1002/mabi.202400286

**Published:** 2024-10-03

**Authors:** Cynthia V Yapto, Keerthana Rajes, Antonia Inselmann, Sven Staufenbiel, Kim N Stolte, Maren Witt, Rainer Haag, Henrik Dommisch, Kerstin Danker

**Affiliations:** ^1^ Institute of Biochemistry Charité – Universitätsmedizin Berlin 10117 Berlin Germany; ^2^ Freie Universität Berlin Institute of Chemistry and Biochemistry 14195 Berlin Germany; ^3^ Charité – Universitätsmedizin Berlin, corporate member of Freie Universität Berlin, Humboldt‐Universität zu Berlin, and Berlin Institute of Health Charitéplatz 1 10117 Berlin Berlin Germany; ^4^ Freie Universität Berlin, Institute of Pharmacy Pharmaceutical Technology 12169 Berlin Germany; ^5^ Department of Periodontology, Oral Medicine and Oral Surgery Charité – Universitätsmedizin Berlin 14197 Berlin Germany

**Keywords:** core‐multishell nanocarrier, dexamethasone, drug delivery, epithelial cells, oral mucosal inflammation

## Abstract

Topical treatment of oral inflammatory diseases is challenging due to the intrinsic physicochemical barriers of the mucosa and the continuous flow of saliva, which dilute drugs and limit their bioavailability. Nanocarrier technology can be an innovative approach to circumvent these problems and thus improve the efficacy of topical drug delivery to the mucosa. Core‐multishell (CMS) nanocarriers are putative delivery systems with high biocompatibility and the ability to adhere to and penetrate the oral mucosa. Ester‐based CMS nanocarriers release the anti‐inflammatory compound dexamethasone (Dx) more efficiently than a conventional cream. Mussel‐inspired functionalization of a CMS nanocarrier with catechol further improves the adhesion of the nanocarrier and may enhance the efficacy of the loaded drugs. In the present study, the properties of the ester‐based CMS 10‐E‐15‐350 nanocarrier (CMS‐NC) are further evaluated in comparison to the catechol‐functionalized variant (CMS‐C_0.08_). While the mucoadhesion of CMS‐NC is inhibited by saliva, CMS‐C_0.08_ exhibits better mucoadhesion in the presence of saliva. Due to the improved adhesion properties, CMS‐C_0.08_ loaded with dexamethasone (Dx‐CMS‐C_0.08_) shows a better anti‐inflammatory effect than Dx‐CMS‐NC when applied dynamically. These results highlight the superiority of CMS‐C_0.08_ over CMS‐NC as an innovative drug delivery system (DDS) for the treatment of oral mucosal diseases.

## Graphical abstract

CMS nanocarriers are delivery systems that can be loaded with both hydrophobic and hydrophilic drugs. Proof of concept is provided in two different models that administration of dexamethasone by CMS‐NC led to a reduction in the TNFα‐induced inflammatory response of the oral mucosa. Catechol‐functionalization of CMS‐NC increases muco‐adhesion and enhances the anti‐inflammatory effect when loaded with dexamethasone after dynamic application. Figure is created with BioRender.

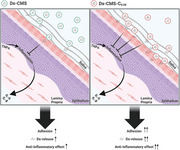

## 1 Introduction

In the oral cavity, numerous inflammatory diseases affect the epithelial surface, compromising the physiological barrier of the oral mucosa. Deterioration of the epithelial surface can affect the patient's health not only locally but also systemically. Several pathological pathways can lead to mucosal inflammation, including microbiologically and auto‐immunologically induced processes such as periodontitis and oral lichen planus.^[^ ^1^ ^]^ For the latter, the available treatment concept is limited to the application of corticosteroids in the form of creams and mouth rinses,^[^ ^2^ ^]^ whereas for periodontitis, the therapy only addresses the microbiological aspect, leaving the complex inflammatory reaction untreated. The extent of oral mucosal destruction depends on the individual's immunological capacity, which may be influenced by age, systemic diseases, and genetic risk factors. Novel anti‐inflammatory therapeutic approaches have been studied by experimental periodontitis in animals.^[^ ^3^, ^4^, ^5^, ^6^ ^]^ Due to the accessibility of oral mucosal tissues, topical application of anti‐inflammatory drugs is highly desired to bypass the liver and thereby, reduce unintended systemic side effects. However, the oral mucosa is separated from the external environment by a ridged physical barrier formed by stratification of epithelial cell layers and tight junctions that prevent mechanical injury and pathogen invasion.^[^ ^7^ ^]^ In addition, saliva and gingival crevicular fluid cover the epithelial surface and provide a constant fluid flow along the tooth surface, respectively, further hindering a microbial invasion into deeper tissue layers. In the same context, these structural conditions also limit the adhesion of drugs to the mucosal surface and thus, their penetration into the epithelial cell layers. In addition, saliva and crevicular fluid dilute drugs, leading to a reduction in bioavailability.^[^ ^3^, ^4^, ^5^ ^]^ The flow and dilution effects of both biological fluids increase under inflammatory conditions.^[^ ^8^, ^9^ ^]^ Therefore, there is a strong medical need for the development of novel delivery systems that overcome these challenges and efficiently deliver anti‐inflammatory agents to their intended site of action.

Here, the emergence of nanotechnology opens new therapeutic options in oral medicine. Nano‐based drug delivery systems have been tested for their potential application as drug carrier systems in the treatment of periodontitis, such as chitosan‐, polylactic‐co‐glycolic acid (PLGA)‐based nanoparticles and PAMAM dendrimers, which can efficiently deliver drugs to the target site due to their size and other physicochemical properties.^[^ ^10^, ^11^ ^]^ For example, chitosan and its derivatives act as permeation enhancers by transiently and reversibly opening tight junctions. They have been shown to increase the transport of hydrophilic compounds across the small intestinal mucosa.^[^ ^12^ ^]^ Chitosan is a non‐toxic, biocompatible and biodegradable material that exhibits both antibacterial and anti‐inflammatory activities.^[^ ^11^ ^]^ Its antibacterial activity is exerted by binding to the negatively charged surface of microorganisms, which disrupts the cell membrane and ultimately leads to cell death.^[^ ^13^ ^]^ An in vitro study demonstrated the antibacterial activity of chitosan against Porphyromonas gingivalis.^[^ ^14^, ^15^ ^]^ The antimicrobial activity of chitosan‐based nanocarriers improved with molecular weight and with chlorhexidine as a cargo.^[^ ^11^, ^14^ ^]^ Another nano‐based drug delivery system tested as an oral mucosal drug delivery system, is dendritic poly(glycerol‐caprolactone) sulfate (dPGS‐PCL), which adheres to and efficiently penetrates the oral mucosa.^[^ ^16^ ^]^ In addition, it has intrinsic anti‐inflammatory activity, which could be used to reduce IL‐8 secretion and thus the inflammation.

Recently, we introduced the concept of CMS nanocarriers as novel drug delivery systems for topical drug application to the oral mucosa, which exhibited good adhesion and penetration properties.^[^ ^17^, ^18^ ^]^ These delivery systems were particularly characterized by their biocompatibility, defined size, high colloidal solubility and ability to incorporate and transport both hydrophilic and hydrophobic compounds.^[^ ^19^ ^]^ In an ex vivo approach using porcine oral mucosa, the dexamethasone‐loaded CMS nanocarrier was shown to release dexamethasone more efficiently than a conventional cream formulation.^[^ ^18^ ^]^ Functionalization of CMS nanocarriers with catechol, a substance used by mussels to adhere to wet surfaces, resulted in stronger adhesion properties than the non‐functionalized version.^[^ ^20^, ^21^, ^22^, ^23^ ^]^

The aim of this study was to evaluate the anti‐inflammatory effect of the dexamethasone‐loaded CMS nanocarrier (Dx‐CMS) compared to the unloaded nanocarrier (CMS‐NC), a dexamethasone solution (Dx) and a conventional dexamethasone cream (Dx‐Cream) in both cell monolayers and a complex 3D model of inflammatory oral mucosa. In addition, their adhesion to the mucosal surface of gingival epithelial cells was evaluated in the presence of human saliva and compared with the mucoadhesive properties of the mussel‐inspired catechol‐functionalized CMS variant.^[^ ^22^, ^24^, ^25^ ^]^ The anti‐inflammatory properties of the catechol‐functionalized nanocarrier were determined after loading with dexamethasone and compared with those of Dx‐CMS under both static and dynamic conditions.

## 2 Results and Discussion

### 2.1 TNFα Upregulated Pro‐Inflammatory Cytokines in Oral Epithelial Cells

Periodontitis as well as oral lichen planus are complex inflammatory diseases characterized by the release of pro‐inflammatory cytokines, such as IL‐6, IL‐8, IL‐1ß, IFN γ and TNFα.^[^ ^7^, ^26^, ^27^ ^]^ Particularly in periodontitis, the dysregulation of proinflammatory cytokines such as TNFα could trigger inflammation leading to tissue and alveolar bone destruction.^[^ ^28^, ^29^ ^]^

**Figure 1:**
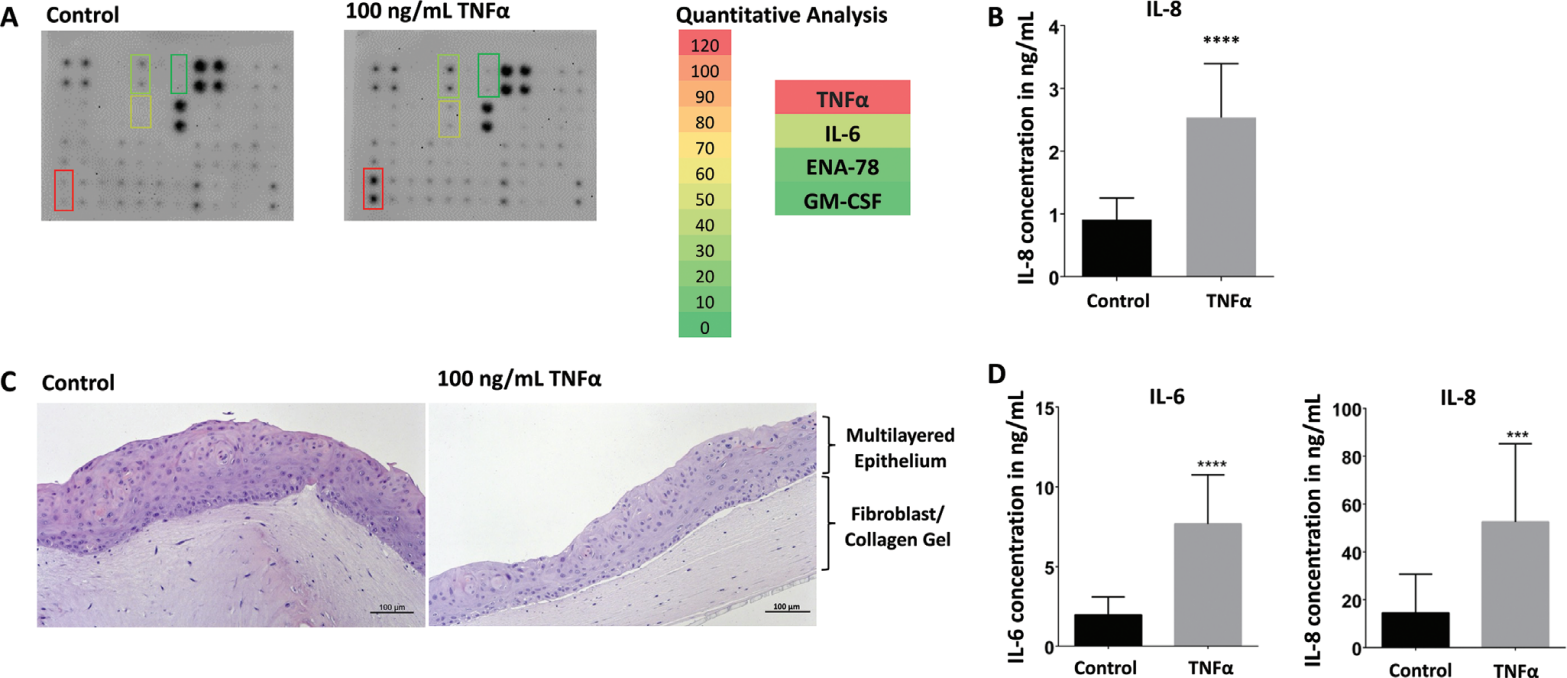
A) Human cytokine antibody arrays incubated with cell supernatants collected after 24 h TNFɑ stimulation on immortalized oral epithelial cells OKG4 and the unstimulated control. The colors indicate the degree of cytokine upregulation. B) The secretion of the pro‐inflammatory cytokine IL‐8 in OKG4 cell monolayers following 24 h of TNFɑ stimulation compared to the secretion observed in untreated cells, determined by ELISA. C) Hematoxylin and eosin staining of 3D oral mucosa models stimulated with 100 ng mL^−1^ TNFɑ (right) or left untreated (left). D) The levels of the pro‐inflammatory cytokines IL‐6 and IL‐8 secreted by the immortalized 3D models after 24 h TNFɑ stimulation and the control. At least three independent experiments were performed (* 0.01 ≤ *p* < 0.05; ** 0.001 ≤ *p* < 0.01; *** 0.0001 ≤ *p* < 0.001; **** *p* < 0.0001).

In this study, the inflammatory effect of TNFα was analyzed using different oral mucosal models consisting of immortalized gingival keratinocytes by monitoring the release of pro‐inflammatory cytokines. A human cytokine antibody array was used for this purpose (**Figure** **1**A). The map that depicts the positions for putative cytokine binding is shown as Table S1 (Supporting Information). After 24 h of TNFα stimulation, inflammatory markers such as IL‐6, ENA‐78 and GM‐CSF were upregulated 37‐, 9‐ and 4‐fold, respectively (Figure 1A).

In the cytokine array, the increase of the chemokine IL‐8 was slightly below the defined threshold of 2 with a fold change of 1.57 (Table S2, Supporting Information). In addition, our previous study showed the increase of IL‐8 during IL‐1ß‐induced inflammation,^[^ ^7^ ^]^ so the concentration of IL‐8 from the cell culture medium of immortalized gingival keratinocytes was additionally determined using the more accurate ELISA analysis. A significant increase in IL‐8 levels was observed after 24 h of TNFα stimulation in cell monolayers (Figure 1B). Thus, TNFα induced an inflammatory response in gingival keratinocytes. A similar response was elicited in these cells in response to IL‐1ß stimulation.^[^ ^7^ ^]^

The effect of TNFα was also tested in a more complex 3D model of human oral mucosa. This model was constructed from immortalized gingival keratinocytes and fibroblasts to mimic the mucosa in its full thickness with the multilayered epithelium and underlying lamina propria (Figure 1C). Previous studies have shown that epithelial cells undergo terminal differentiation and form barriers that can be used to test nano‐sized drug delivery systems.^[^ ^16^, ^17^ ^]^ In this study, hematoxylin and eosin staining of paraffin‐embedded models was performed on thin sections to evaluate the histological differences between untreated and TNFα‐treated 3D models (Figure 1C). While the untreated model (left) showed a multilayered keratinized epithelium, TNFα treatment (right) resulted in a reduced adhesion of the upper epithelial layers to the collagen/fibroblast gel and a thinner matrix. To study the inflammatory response, these 3D models were used for further analysis. As shown for the cell monolayers, TNFα induced the elevation of IL‐6 and IL‐8 levels in the 3D oral mucosa model after 24 h compared to the control model (Figure 1D). These data demonstrated that TNFα can induce an inflammatory response in both models of oral mucosa examined, the differentiated cell monolayer and the full‐thickness 3D model. These data confirmed the study by Witt and coworkers, which showed an upregulation of the classical proinflammatory cytokines IL‐6 and IL‐8.^[^ ^16^ ^]^ IL‐6 and IL‐8, here induced by TNFα, are secreted by epithelial cells of the oral mucosa as a first line of defense in inflammatory diseases such as periodontitis, suggesting their important role as targets for the treatment of oral diseases and were therefore considered suitable inflammatory markers to study anti‐inflammatory effects.^[^ ^30^, ^31^ ^]^ Thus, stimulation with the important inflammatory mediator TNFα leads to inflammatory models in which the influence on a periodontitis‐like inflammatory process can be monitored.^[^ ^30^ ^]^

### 2.2 Dx‐CMS Reduced IL‐6 and IL‐8 Levels in Oral Epithelial Cells and 3D Models

CMS‐NC was previously characterized for its potential use as an oral mucosal drug delivery system. In addition to its biocompatibility, CMS‐NC could adhere to cell monolayers and penetrate the epithelial layer of ex vivo porcine oral mucosa and the epithelium of the 3D oral full‐thickness model within minutes.^[^ ^17^ ^]^ Another study showed that CMS‐NC could release the synthetic anti‐inflammatory glucocorticoid dexamethasone more efficiently than a conventional cream formulation in both the masticatory and buccal porcine oral mucosa.^[^ ^18^ ^]^ Dexamethasone is commonly used in clinical practice for the treatment of inflammatory diseases.^[^ ^32^, ^33^ ^]^ In particular, in oral lichen planus, dexamethasone treatment was found to significantly reduce salivary levels of pro‐inflammatory cytokines.^[^ ^34^ ^]^ Dexamethasone exerts its anti‐inflammatory activity by binding to the glucocorticoid receptor (GR). This induces translocation of GR to the nucleus, where it can bind to the glucocorticoid response element or to other transcription factors, such as nuclear factor‐κB (NF‐κB) and activator protein 1 (AP‐1).^[^ ^33^, ^35^ ^]^ Binding of the GC/GR complex to NF‐κB and AP‐1, key transcription factors in inflammation, modulates their activity, resulting in suppression of pro‐inflammatory genes and dampening of inflammation.^[^ ^35^ ^]^ The significant reduction of pro‐inflammatory cytokines such as IL‐1ß, TNFα, IL‐6 and IL‐8 when cells were pretreated with dexamethasone was also shown.^[^ ^36^ ^]^

**Figure 2:**
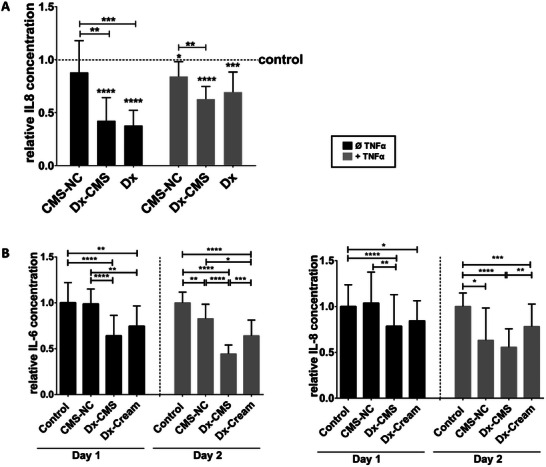
A) Analysis of IL‐8 levels in OKG4 cells after treatment with the Dx, Dx‐CMS, or CMS‐NC in the absence (black bar) or presence (gray bar) of TNFɑ. B) Analysis of IL‐6 (left panel) and IL‐8 (right panel) concentrations in 3D models composed of immortalized gingival keratinocytes and fibroblasts after treatment with CMS‐NC, Dx‐CMS or Dx‐cream in the absence (day 1; black bar) and presence (day 2; gray bar) of TNFɑ. All experiments were performed in at least three independent biological replicates. ELISA experiments were performed with at least 3 technical replicates each. Error bars represent the standard deviation (SD) (* 0.01 ≤ *p* < 0.05; ** 0.001 ≤ *p* < 0.01; *** *p* < 0.001; **** *p* < 0.0001).

Using differentiated OKG4 cells, we investigated whether Dx encapsulated in CMS‐NC (Dx‐CMS) also had a better anti‐inflammatory effect than Dx dissolved in ethanol. TNFα‐induced IL‐6 and IL‐8 levels were used as indicators and compared with IL‐6 and IL‐8 levels of untreated cells. To investigate the anti‐inflammatory effect, cells were treated with Dx, Dx‐CMS or unloaded CMS‐NC for 48 h (**Figure** **2**A; black bar) or pretreated with Dx, Dx‐CMS or unloaded CMS‐NC for 24 h and then stimulated with TNFα for another 24 h (Figure 2A; gray bar). Cell supernatants collected after 48 h were analyzed for IL‐8 levels and compared to the IL‐8 levels of the respective solvent control, which was set to 1. Cells treated with Dx or Dx‐CMS in the absence of TNFα secreted significantly less IL‐8 than the corresponding controls, whereas treatment with unloaded CMS‐NC had no significant effect on IL‐8 secretion. Treatment with Dx‐CMS and Dx inhibited IL‐8 secretion to a similar extent (Figure 2A (black bar)). However, compared to CMS‐NC treatment, both were significantly more effective in reducing IL‐8 levels.

Similar effects were observed in TNFα‐stimulated cells but were less pronounced (Figure 2A; gray bars). Both Dx and Dx‐CMS significantly reduced IL‐8 secretion and showed comparable anti‐inflammatory effects. However, compared to CMS‐NC treatment, both showed significantly greater efficacy in reducing IL‐8 levels. This can be attributed to the anti‐inflammatory effect of dexamethasone in both non‐inflamed (Figure 2A; black bar) and inflamed conditions (Figure 2A; gray bar). After 48 hours incubation with the unloaded CMS‐NC, a slight but significant decrease in IL‐8 release was observed compared to the control (dotted line).

In conclusion, Dx and Dx‐CMS showed comparable anti‐inflammatory effects in both healthy and inflamed conditions. The efficacy of the hydrophobic compound dexamethasone alone may be due to its solvent vehicle ethanol. Ethanol has been described as a permeation enhancer that increases the permeability of the lipid bilayer of the plasma membrane by extracting skin lipids and increasing the mobility of lipid chains, which may facilitate the transport of dexamethasone into cells.^[^ ^37^ ^]^ Given that an ethanolic solution is not optimal for application to the oral mucosa, the CMS nanocarrier appears to be a promising option for transporting drugs to the mucosa, as demonstrated here with dexamethasone as a model compound. Proof‐of‐concept was provided that the Dx‐CMS drug delivery system has anti‐inflammatory activity in oral epithelial cells. These results are consistent with data from a previous study showing that Dx can be released from the CMS nanocarrier in the oral mucosa using EPR spectroscopy.^[^ ^18^ ^]^ Since studies on cell monolayers have limited predictive power, the anti‐inflammatory effect of the DDS was also evaluated on the more complex full‐thickness 3D model of the oral mucosa constructed from immortalized keratinocytes and fibroblasts. These models more accurately reflect the in vivo situation. Dx‐CMS, unloaded CMS‐NC and a cream formulation containing Dx (Dx‐cream), which is commonly used to treat inflammation in the oral cavity,^[^ ^18^ ^]^ was applied to the 3D models on day 1 (Figure 2B; black bars). Untreated 3D models served as controls. On day 2, the 3D models were stimulated with TNFα via the medium for 24 h (Figure 2B; gray bars). Cell culture media were collected every 24 h and IL‐6 and IL‐8 levels were determined using ELISA. The anti‐inflammatory effect of the various treatment approaches was evaluated by setting the concentrations of IL‐6 and IL‐8 to 1 in the untreated model after 24 hours and in the TNFα‐stimulated models after 48 hours, respectively. In the 3D model, Dx‐CMS and Dx‐cream were shown to significantly reduce IL‐6 and IL‐8 levels under both non‐inflammatory (Figure 2B; black bar) and inflammatory (Figure 2B; gray bar) conditions. In comparison to the treatment with Dx cream, treatment with Dx‐CMS showed a significant reduction in IL‐6 and IL‐8 levels under inflamed conditions. This suggests that dexamethasone formulated in the drug delivery system is more effective than in the cream, as postulated in the study by Jager and co‐workers.^[^ ^18^ ^]^ As demonstrated in the 2D cell culture (Figure 2A), the unloaded CMS‐NC leads to a slight but significant reduction in the release of both cytokines after 48 hours also in the 3D model.

The findings that the concentration of pro‐inflammatory cytokines also decreases after a longer incubation period with the unloaded nanocarrier could be related to the fact that the nanocarrier absorbs cytokine molecules with its free valencies, which then could not be detected in the ELISA. An intrinsic anti‐inflammatory activity, as described for other delivery systems is rather unlikely and has not been observed when applied to other tissues, such as the skin.^[^ ^16^, ^38^ ^]^

Dx‐CMS was also tested in a 3D model prepared from primary human keratinocytes and fibroblasts from a healthy donor. In this model, Dx‐CMS further reduced the levels of IL‐6 and IL‐8, which was observed in both the non‐inflamed and inflamed conditions. The effect of Dx‐CMS was also more pronounced compared to Dx cream (Figure S1, Supporting Information).

### 2.3 Catechol Functionalization of the CMS Nanocarrier Improved Muco‐Adhesion in the Presence of Saliva

**Figure 3:**
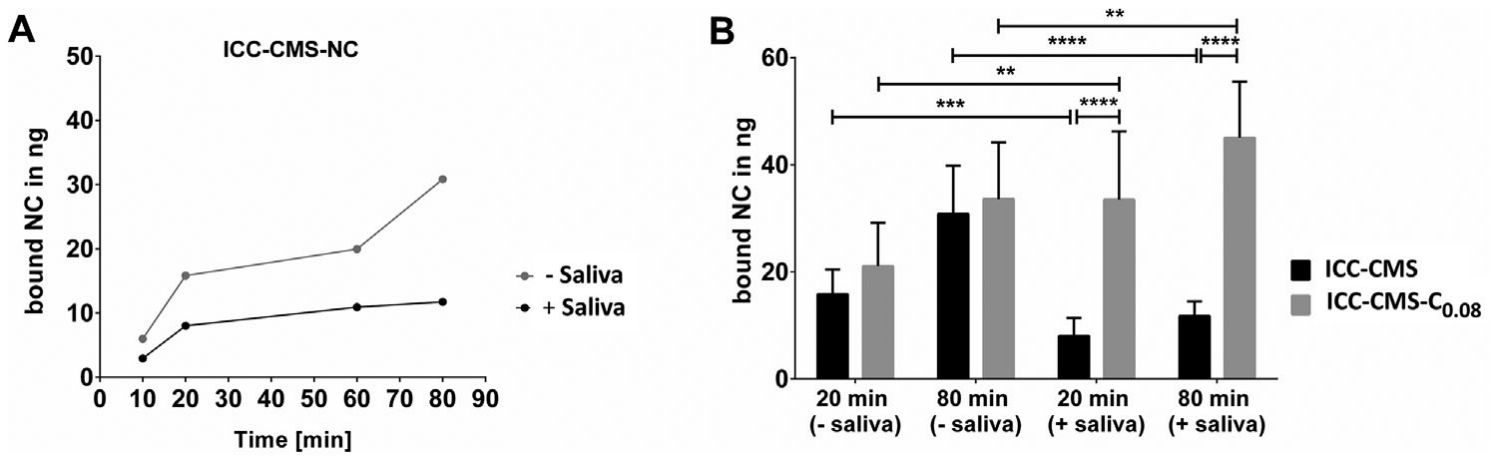
A) Time‐dependent adhesion of ICC‐CMS‐NC in the presence or absence of saliva after 10 min, 20 min, 60 min, and 80 min of incubation. B) Quantification of CMS nanocarriers bound to cell monolayers after 20 min and 80 min, ICC‐CMS‐NC (black bar) and ICC‐CMS‐C0.08 (gray bar), in the presence or absence of saliva. Three independent experiments were performed on differentiated OKG4 cell monolayers (* 0.01 ≤ *p* < 0.05; ** 0.001 ≤ *p* < 0.01; *** 0.0001 ≤ *p* < 0.001; **** *p* < 0.0001).

Successful application of DDS to mucosa in general and to oral mucosa in particular, requires adequate adhesion of the DDS to the mucosa (so‐called muco‐adhesion), which aims to prolong the retention time of the DDS on the mucosa and thus increase the bioavailability of the drug.^[^ ^39^, ^40^ ^]^ In previous studies, we have shown that CMS nanocarriers adhere to ex vivo models of masticatory and buccal mucosa.^[^ ^17^, ^18^ ^]^ However, all previous studies of CMS nanocarriers on muco‐adhesion have neglected the role of saliva, which is a challenging obstacle for topical therapy on the oral mucosa. The constant flow of saliva not only dilutes the drug but also acts as an additional barrier for the DDS, making adhesion to the mucosa more difficult. Therefore, we further characterized the adhesion properties of the CMS nanocarrier in the presence of saliva. The experiments were conducted using cell monolayers derived from OKG4 cells that had undergone differentiation. Prior to incubation with the CMS nanocarriers, the cell medium was replaced with sterile‐filtered saliva or fresh cell culture medium. The cells were then incubated for 20 min. Without removing the saliva or cell culture medium, indocarbocyanine (ICC)‐labeled CMS nanocarrier was added to the wet cell monolayers for the indicated time points (**Figure** **3**A). Cells were then washed to remove unbound nanocarrier. ICC fluorescence was determined using a fluorescence microplate reader. The values obtained were normalized to the nanocarrier loading control, and the amount of bound nanocarriers was calculated. In the absence of saliva, the amount of bound nanocarrier (ICC‐CMS‐NC) exhibited a gradual increase over time. The addition of saliva resulted in a reduction in adhesion at all time points compared to the adhesion observed in the absence of saliva, and binding of the nanocarrier was found to be saturated after only 20 min (Figure 3A). Thus, saliva appears to inhibit the adhesion of the CMS nanocarrier to the mucosal surface.

We have recently introduced a CMS variant designed to improve muco‐adhesion. This variant combines the advantages of the core‐multishell system with the ability to adhere to wet surfaces by functionalizing the outer shell of the CMS nanocarrier with catechol groups.^[^ ^20^ ^]^ Catechol is a side chain of the amino acid, L‐3,4‐dihydroxyphenylalanine and has been described to mediate mussel adhesion to wet surfaces.^[^ ^21^, ^22^, ^23^ ^]^ Using two complementary adhesion assays, we demonstrated that functionalization of CMS with catechol improved the adhesive properties of the CMS nanocarriers to mucosal surfaces. The 8% catechol functionalization showed the strongest binding to cell monolayers of differentiated gingival keratinocytes.^[^ ^20^ ^]^ To test the adhesive properties of the catechol‐functionalized nanocarrier in more detail, muco‐adhesion in the presence and absence of saliva was examined and directly compared with the adhesive properties of the non‐functionalized nanocarrier. For this purpose, the time points 20 min and 80 min were chosen, where saliva had a profound influence on the adhesion of the non‐functionalized nanocarrier (Figure 3A).

In the absence of saliva, the adhesion of the two types of nanocarrier to the gingival epithelial cells was comparable. The amount of bound ICC‐CMS‐NC and ICC‐CMS‐C_0.08_ each increased over time, which was also observed in a previous study.^[^ ^20^ ^]^ In the presence of saliva, the adhesion of the non‐functionalized CMS nanocarrier was highly significantly reduced (Figure 3B). In contrast, the adhesion of the catechol‐functionalized nanocarrier was significantly increased in the presence of saliva. This increase may be due to irreversible cross‐linking of catechol residues with salivary mucins mediated by the irreversible covalent bonds of the o‐quinone form of catechol with various functional groups, such as thiol and amine groups, present in the proteoglycan structure of mucin.^[^ ^22^, ^41^, ^42^, ^43^, ^44^ ^]^ Mussel‐inspired catechol functionalization has been described in several studies to improve the adhesion properties of other bio‐adhesive polymers, e.g., chitosan‐based polymers in wet environments and therefore, appears to be a useful tool to generate efficient mucoadhesive drug delivery systems.^[^ ^22^, ^24^, ^25^ ^]^ Consistent with these studies, our results confirmed that catechol functionalization also improved the adhesion properties of the CMS nanocarrier to saliva‐coated mucosal surfaces and thus, could be an option for drug delivery in the oral mucosa.

### 2.4 Catechol Functionalization of the CMS Nanocarrier did not Affect the Anti‐Inflammatory Capacity of the Dx‐CMS System

**Figure 4:**
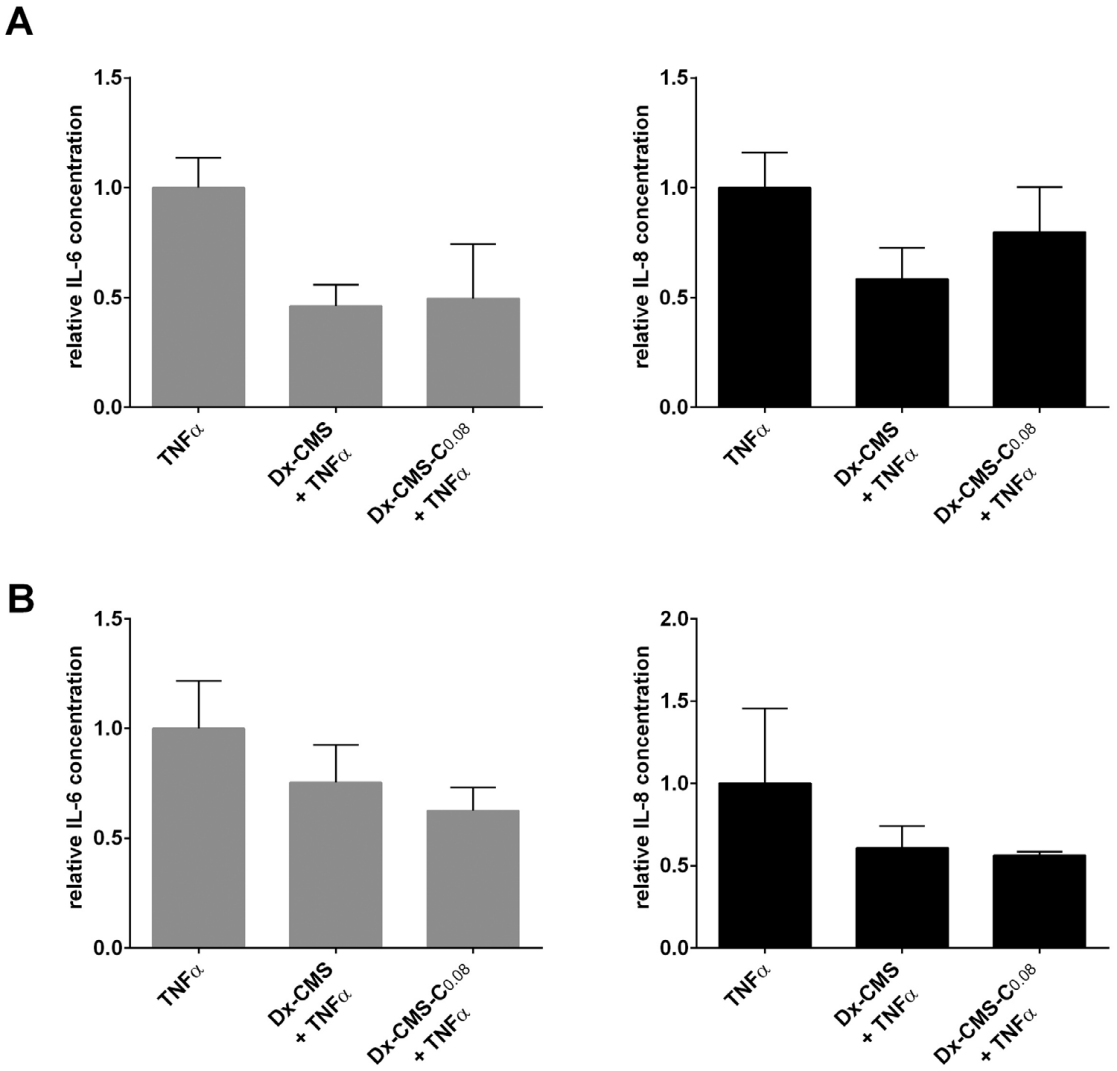
Effect of Dx‐CMS‐C_0.08_ and Dx‐CMS in TNFɑ‐stimulated 3D models A) composed of immortalized cells and B) composed of primary cells on IL‐6 (left) and IL‐8 (right) secretion. At least two independent experiments were performed with 3D models composed of immortalized cells. One experiment was performed with 3D composed of primary cells. All ELISA experiments were performed with at least 3 technical replicates each. The error bars represent the standard deviation (SD).

We further investigated whether catechol modification of the CMS surface might have a negative influence on the anti‐inflammatory activity of the Dx‐CMS system. Therefore, the release of IL‐6 and IL‐8 from Dx‐CMS‐C_0.08_ and Dx‐CMS‐treated 3D models was analyzed and compared in the presence of TNFα (**Figure** **4**). The 3D models were pretreated with Dx‐CMS‐C_0.08_ or Dx‐CMS for 24 h or left untreated. The next day, the 3D models were stimulated with TNFα from the medium below for the next 24 h by replacing the metabolized medium with fresh medium containing TNFα. The cell medium was then collected and subjected to ELISA analysis.

ELISA analysis showed that both Dx‐CMS and Dx‐CMS‐C_0.08_ markedly decreased the levels of IL‐6 and IL‐8 in the medium of the 3D cell models to a similar extent in both immortalized and primary 3D models (Figure 4A,B). Compared to the results in Figure S1 (Supporting Information) (gray bar), the anti‐inflammatory effect of Dx‐CMS on IL‐6 and IL‐8 was less pronounced. This may be due to differences in donor response, as two different donors were used to generate both 3D primary models.

Overall, it can be concluded that both nanocarriers, Dx‐CMS and Dx‐CMS‐C_0.08_, possess similar anti‐inflammatory efficacy when administered statically, and thus, catechol functionalization did not affect the anti‐inflammatory capacity of the Dx‐CMS system.

### 2.5 Dx‐CMS‐C_0.08_ Exerted a Superior Anti‐Inflammatory Effect after Dynamic Application in Oral Epithelial Cells

The similar efficacy of the dexamethasone‐loaded catechol‐functionalized and unfunctionalized CMS variants can be attributed to the long incubation times of 24 h and 48 h, respectively, with the same dexamethasone loading. We hypothesize that the administered nanocarriers are completely taken up by the cells via endocytosis during this time.^[^ ^45^ ^]^ The superiority of the mucoadhesive nanocarrier should therefore become more evident following a short and dynamic application on the mucosal surface, which is a crucial prerequisite for topical application on the oral mucosa.

**Figure 5:**
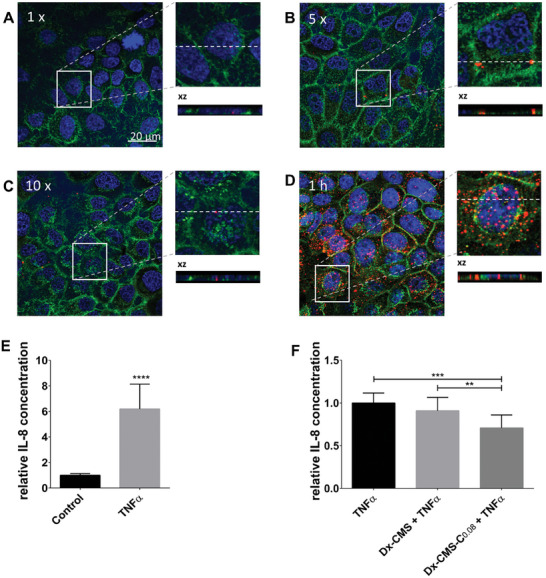
Visualization of ICC‐CMS‐NC attached to differentiated OKG4 cells after the nanocarrier was passed A) once, B) five times, or C) ten times along the cell monolayer. As a positive control, differentiated cells were incubated with the ICC‐coupled CMS nanocarrier for D) 1 h. All images were taken with a confocal microscope (LSM700MAT; Zeiss) at 63x magnification; scale bar: 20 µm. E) Analysis of IL‐8 after 21 h TNFɑ stimulation or without stimulation (control) using ELISA F) Comparison of the anti‐inflammatory capacity of Dx‐CMS and Dx‐CMS‐C_0.08_ after dynamic application. At least three independent experiments were performed on differentiated OKG4 cells (* 0.01 ≤ *p* < 0.05; ** 0.001 ≤ *p* < 0.01; *** 0.0001 ≤ *p* < 0.001; **** *p* < 0.0001).

The concept of the dynamic adhesion assay has already been presented in a previous study and illustrated again in **Figure** **5** (A‐D) for ICC‐coupled CMS‐NC (ICC‐CMS‐NC).^[^ ^20^ ^]^ ICC‐CMS‐NC at a concentration of 50 µg/mL was dynamically passed once, five times or ten times along an angulated cell monolayer. The attached nanocarrier was monitored by confocal microscopy. For orientation, cell membranes were counterstained with Alexa Fluor 488‐conjugated wheat germ agglutinin (WGA) and cell nuclei were counterstained with DAPI. Weak binding of ICC‐CMS‐NC was observed after one time of rinsing (Figure 5A). After five (Figure 5B) and ten (Figure 5C) rinses, ICC‐CMS‐NC signals increased. Xz projections taken from the xy projection (Figure 5A–D; dashed lines) showed localization of ICC‐CMS‐NC on and in epithelial cells under these conditions (Figure 5A–C). Cells treated with the solvent are shown in Figure S2 (Supporting Information). Compared to dynamic application of ICC‐CMS‐NC on the cell monolayer, adhesion under static conditions for one hour resulted in visibly higher ICC‐CMS‐NC signals and distinct cellular uptake of the nanocarrier with cytosolic localization, as shown by xy and xz projections (Figure 5D). Additional xz projections of each treatment are shown in Figure S3 (Supporting Information).

Using this dynamic approach, we have recently shown that the catechol‐functionalized CMS nanocarrier loaded with Nile Red binds more strongly to differentiated mucosal cells and releases its cargo Nile Red better than its catechol‐free counterpart.^[^ ^20^ ^]^ We therefore hypothesized that improved adhesion and uptake into cells should result in improved anti‐inflammatory effects. Therefore, the dynamic adhesion assay with Dx‐CMS and Dx‐CMS‐C_0.08_ nanocarriers was performed by passing the nanocarriers five times over cell monolayers of differentiated oral epithelial cells. The cells were then re‐cultured and stimulated with TNFα for an additional 21 h or left untreated. The inflammatory response was then measured by IL‐8 levels using ELISA (Figure 5E,F). While stimulation with TNFα highly significantly increased IL‐8 levels in oral epithelial cells (Figure 5E), treatment with Dx‐CMS reduced the relative concentration of IL‐8 to 0.91 compared to TNFα‐stimulated cells which was set to 1. This reduction was only a trend without reaching statistical significance. However, Dx‐CMS‐C_0.08_ was more effective and significantly reduced IL‐8 release to 0.71 compared to the TNFα stimulated cell monolayer.

Taken together, the improved muco‐adhesion of the CMS‐C_0.08_ nanocarrier appears to have resulted in enhanced cellular uptake, which in turn led to a more efficacious drug release and a more pronounced anti‐inflammatory effect of dexamethasone.^[^ ^20^ ^]^

## 3 Conclusion

Previous studies have evaluated the potential application CMS nanocarriers as drug delivery systems in the skin and oral mucosa. These studies have assessed the biocompatibility, penetration, muco‐adhesion, and drug release properties of these nanocarriers.^[^ ^17^, ^18^, ^19^ ^]^ However, in the dynamic environment of the oral cavity, where saliva is continuously flowing, nanocarriers that adhere to the mucosal surface with high affinity are required. Catechol functionalization has been presented as a viable option for improving adhesion in the presence of saliva. When applied dynamically, functionalization of the CMS nanocarrier with catechol residues resulted in a significant reduction in IL‐8 release. These results suggest that the CMS‐C_0.08_ nanocarrier loaded with Dx or other anti‐inflammatory drugs effectively reduces epithelial inflammation. It is therefore conceivable that this formulation could be used in an anti‐inflammatory mouthwash, as this delivery system meets the critical requirements for use on the oral mucosa. This innovative approach may have applications in the treatment of various oral inflammatory conditions.

## 4 Experimental Section

### Reagents

ICC‐OH was kindly provided by PolyAn GmbH. Dimethylaminopyridine (DMAP) was purchased from Acros Organics (Fair Lawn, NJ, USA). Sephadex G‐25 was purchased from VWR/cytiva. Core‐multishell nanocarriers, derived from the 10‐E‐15‐350 nanocarrier were synthesized and include CMS‐NC, ICC‐CMS‐NC, and Dx‐CMS. These nanocarriers were synthesized according to previously described protocols.^[^ ^19^, ^20^, ^46^ ^]^ A concentration of 50 µg mL^−1^ of each nanocarrier was used for all experiments.

### Dexamethasone‐Loaded CMS‐NC Carrier

For dexamethasone loading, CMS‐NC (5 mg mL^−1^) was dissolved in distilled water (1 mL). Dexamethasone (50 wt%) was added, and the solution was stirred for 18 h before being filtered through a 0.45 µm RC filter. The drug content was then determined by HPLC measurements, which showed dexamethasone loadings between 3.3 – 8 wt%. The remaining solvent was then removed to redissolve the residue in DermaLife K cell culture medium, resulting in a final concentration of 5 mg mL^−1^ for the nanocarrier.

### Dexamethasone‐Loaded CMS‐C_0.08_ Nanocarrier

The synthesis of CMS‐C_0.08_ has been described previously.^[^ ^20^ ^]^ To load dexamethasone into the carrier, CMS‐C_0.08_ was deprotected by stirring in methanol at 70 °C for 2 h. After removing the solvent, it was redissolved in distilled water at a concentration of 5 mg ml^−1^. Dexamethasone of 50 wt% was added, and the solution was stirred for 18 h. The filtered solution was passed through an 0.45 µm RC filter and the drug content determined by HPLC measurements showed dexamethasone loadings of 2.3 – 3.4 wt%. The remaining solvent was evaporated to redissolve the residue in DermaLife K cell culture medium, resulting in a final nanocarrier concentration of 5 mg mL^−1^.

### Dexamethasone Cream

The dexamethasone cream was prepared as described in the previous study, where dexamethasone (0.18 mg) was formulated in Macrogol ointment (1 g) consisting of Macrogol 400 (92 wt%), Macrogol 1500 (4 wt%), and Macrogol 4000 (4 wt%).^[^ ^18^ ^]^ The dexamethasone cream was resuspended in DermaLife K medium and then filtered through a 0.45 µm filter.

### ICC‐Labeled CMS‐C_0.08_ Nanocarrier

The indocarbocyanine (ICC)‐coupled CMS nanocarriers were synthesized by adding ICC‐OH (0.9 mg; ≈ 2 molecules per carrier) to the respective carrier (25 mg) in dry dimethylformamide (DMF) (3 mL). dicyclohexylcarbodiimide (DCC) (0.4 mg) and DMAP (0.2 mg) were added under ice cooling. The reaction mixture was stirred at room temperature for 24 h and then purified on a sephadex G‐25 column. For experiments, the solvent was removed from the CMS‐ICC carrier solution and the residue was redissolved in DermaLife K cell culture medium to achieve a final concentration of 5 mg mL^−1^.

### High‐Performance Liquid Chromatography (HPLC)

HPLC for the analysis of dexamethasone content was performed on a Knauer Smartline‐HPLC system with an internal UV absorption detector (λ = 254 nm), equipped with a Gemini RP C18 column (Phenomenex, 250 nm × 4.6 mm, particle size: 5 µm) and run with acetonitrile as mobile phase at a flow rate of 1.0 mL mi^−1^n under an isocratic regime. Data were analyzed using ChromGate software (Knauer, Berlin, Germany). For concentration determination, dexamethasone‐loaded CMS nanocarriers were lyophilized and redissolved in acetonitrile to extract dexamethasone. The concentration was determined using a dexamethasone calibration curve (Figure S4, Supporting Information).

### Cell Culture and Inflammatory in Vitro Models

The immortalized human gingival epithelial cells OKG4/bmi1/TERT (OKG4; kindly provided by Susan Gibbs, Amsterdam) were used for cell monolayer experiments.^[^ ^47^, ^48^ ^]^ Primary and immortalized gingival epithelial cells and fibroblasts were used for 3D cell cultures. Primary gingival epithelial cells (GECs) and fibroblasts (GFs) were prepared from gingival tissue samples obtained from tooth extractions and kindly provided by the Department of Periodontology, Oral Medicine and Oral Surgery (Charité – Universitätsmedizin Berlin), and it was approved by the Institutional Review Board (EA/185/16). All donors were informed prior to tissue collection and provided written informed consent. All experiments were performed in accordance with good clinical/laboratory practice (GCP/GLP) guidelines and the WHO Declaration of Helsinki 1964, last updated in Fortaleza 2013 (64^th^ WMA General Assembly, Brazil, October 2013).

Immortalized epithelial cells were cultured in DermaLife K medium (LifeLine Cell Technology, Troisdorf, Germany) supplemented with penicillin/streptomycin (1%) (PAN Biotech, Aidenbach, Germany) in the presence of Ca^2+^ (60 µM or 1.4 mM, respectively) (Merck, Darmstadt, Germany) as indicated. Cells were grown on collagen IV‐coated plates and flasks (20 µg/mL) at 37 °C and 5% CO_2_. GECs were additionally cultured with amphotericin B (1%) (Biochrom, Berlin, Germany). Fibroblasts were cultured in DMEM (Corning, NY, USA) supplemented with FCS (10%) (Biochrom, Berlin, Germany). Like GECs, GFs were cultured in amphotericin B (1%) (Biochrom, Berlin, Germany). The medium was changed every 2–3 days.

To assess the anti‐inflammatory effect on cell monolayers, cells were seeded in 6‐well plates (Corning, Corning, NY, USA) at a density of 2.5 × 10^5^ per well. At 80% confluence, cells were cultured in DermaLife K medium containing 1.4 mM Ca^2+^ for 24 h. Then, cells were preincubated for another 24 h with CMS‐NC, Dx‐CMS, Dx and the respective vehicle controls (PBS, water, and ethanol; AppliChem GmbH, Darmstadt, Germany). Subsequently, 100 ng mL^−1^ TNFα (ImmunoTools, Friesoythe, Germany) was added to the medium and incubated for another 24 h, after which the medium was collected, centrifuged at 2,000 rpm for 3 minutes and stored at −20 °C until ELISA analysis.

### 4.1 Culture of In Vitro Organotypic Mucosal Equivalents and 3D Inflammation Models

Organotypic cell culture of 3D models were generated as previously described.^[^ ^16^, ^17^ ^]^ Briefly, type I bovine collagen solution (1.2 mL) (6 mg mL^−1^; Nutragen, Advanced BioMatrix, San Diego, CA, USA) was mixed with 10x HBSS (300 µl) (Gibco, Waltham, MA, USA) and 2N sodium hydroxide (100 µL) (AppliChem, Darmstadt, Germany). To this solution, FCS (300 µl) (Biochrom, Berlin, Germany) containing fibroblast cells (3 × 10^5^ cells mL^−1^) and sterile H_2_O (1.125 mL) were added. Then, the fibroblast‐collagen I mixture (2.5 mL) was transferred to a Millicell culture plate insert (24 mm diameter, pore size: 0.4 µm; Corning Costar, Corning, NY. USA) placed in 6‐well plates (Corning, Corning, NY, USA). The samples were incubated in a humidity chamber at 37 °C for at least 2 h, and the polymerized fibroblast‐collagen I gel was cultured in DMEM with FCS (10%) at 37 °C in a 5% CO_2_ atmosphere. The next day, immortalized (OKG4) or primary gingival keratinocytes (GEC) (4.2 × 10^6^) were placed on the fibroblast‐collagen I gel, with fibroblasts supplied by DMEM in the well and gingival keratinocytes supplied by the insert with DermaLife K medium containing 60 µM Ca^2+^. The co‐culture was incubated at 37 °C in a 5% CO_2_ atmosphere. After the gingival keratinocytes reached confluence, the 3D oral mucosa equivalent was airlifted. The medium in the well was then replaced with DermaLife K medium containing 1.4 mM Ca^2+^ after removing the insert medium. The 3D oral mucosal equivalent was cultured for an additional 9 days with regular bi‐daily medium replacement. To mimic the moist oral mucosal surface, cell media (100 µL) were applied to the equivalent. 9 days after the airlift, the 3D models were embedded in freezing medium (Tissue‐Tek O.C.T. Compound, Sakura Finetek, Alphen aan den Rijn, The Netherlands). Microsections of 7 µm thickness were cut with a freezing microtome (Frigocut 2800N, Leica, Wetzlar, Germany) and fixed with PFA) 4%) in PBS. The microsections were placed on a microscope slide (Superfrost Plus, Menzel GmbH & Co KG. Braunschweig, Germany) and stored at −20 °C until further analysis.

To investigate an inflammatory response, the model was extended to an inflammatory model by administration of 100 ng ml^−1^ TNFα as indicated. Seven days after airlifting the oral mucosal equivalent, Dx‐cream, Dx‐CMS or Dx‐CMS‐C_0.08_ (each 200 µL) was applied to the surface of the 3D models for 24 hours to determine the anti‐inflammatory effect. The next day, the model was stimulated with TNFα‐containing media (100 ng mL^−1^; ImmunoTools, Friesoythe, Germany), replacing the medium in the well. Cell media collected from the wells every 24 h were stored at −20 °C until ELISA.

### Human Cytokine Antibody Array of TNFα‐Treated Cells

A human cytokine antibody array (Abcam, Cambridge, UK) was used to detect 42 different cytokines (Table S1, Supporting Information). OKG4 cells (2.5 × 10^5^) were seeded in one well of 6‐well plate (Corning, NY, USA). After reaching 80% confluence, the cells were cultured in DermaLife K medium containing 1.4 mM Ca^2+^ for 24 h, followed by stimulation with 100 ng mL^−1^ TNFα for the next 24 h. Cell supernatants were collected, spun at 10,000 rpm for 10 min and stored at −20 °C until further use. Two independent experiments were performed.

The cytokine antibody array was performed according to the manufacturer's instructions (Abcam, Cambridge, UK). Each dot on the array represents the presence of a specific cytokine. The exact positions of all cytokines were listed in Table S1 (Supporting Information). Signals were detected and visualized using a VersaDoc 4000 MP along with QuantityOne 4.6.5 software (BioRad Laboratories, Hercules, CA, USA). ImageJ's Protein Array Analyzer plug‐in was used to quantify the signals. Quantitative analysis of the cytokine array was performed to determine the fold change of up‐ and downregulated cytokines. Only fold changes of < 0.5 and > 2 were considered as downregulation and upregulation, respectively. To avoid false positive signals, signal intensities less than 10% of the positive control were excluded from evaluation.

### IL‐6‐ and IL‐8‐Specific ELISA

A sandwich‐ELISA (ImmunoTools, Friesoythe, Germany) was performed to measure the concentrations of IL‐6 and IL‐8. The cell supernatant was collected and working solution (100 µL) was used per well according to the manufacturer's instructions.

### Test for Muco‐Adhesion of Nanocarriers to Differentiated Gingival Cells Under Dynamic Conditions

OKG4 cells (3.5 × 10^4^) per well were seeded on coverslips (13 mm diameter; Karl Hecht GmbH & Co. KG, Sondheim vor der Rhön, Germany) in a 24‐well plate (Corning, Corning, NY, USA) and cultured in DermaLife K medium with 60 µM Ca^2+^. After reaching 80% confluence, the cells were cultured in DermaLife K medium with 1.4 mM Ca^2+^ for the next 24 h. To assess the adhesion of the CMS nanocarrier under dynamic flow, coverslips coated with cells were removed from the well and transferred to a 35 mm diameter Petri dish (Corning, Corning, NY, USA). The dish was then placed on an inclined fixture as previously described.^[^ ^20^ ^]^ Cells were washed with PBS and then exposed to ICC‐coupled CMS‐NC (50 µg/mL) once, five times, or ten times at a steady flow rate, simulating a mouthwash. The treated cell monolayers were rinsed with PBS and then fixed with PFA (4%) in PBS (Carl Roth GmbH, Karlsruhe, Germany). As a negative control, cells were incubated with medium containing water as a solvent control and as a positive control, cells were incubated with ICC‐coupled CMS‐NC (50 µg mL^−1^) for 1 h. For fluorescence staining, DAPI (Sigma‐Aldrich; D9542, St. Louis, MO, USA) was used to visualize nuclei, and AlexaFluor 488‐conjugated wheat germ agglutinin (WGA) (Invitrogen, Waltham, MA, USA) was used to stain cell membranes. Adhesion of ICC‐coupled CMS‐NC (red) to the cell monolayer was captured in a Z‐stack using LSM700MAT (Zeiss, Wetzlar, Germany) and analyzed using ImageJ (Wayne Rasband, National Institutes of Health, USA, version 1.52t). XZ projections were generated using ZEN version 3.4 (blue edition; Carl Zeiss Microscopy GmbH). Three independent experiments were performed.

To investigate the anti‐inflammatory effect of dexamethasone‐loaded CMS nanocarriers after dynamic application, the dexamethasone‐loaded CMS nanocarriers were passed along the cell monolayers five times. The cells were then washed with PBS and further cultured at 37 °C and 5% CO_2_. After three hours of re‐cultivation, the cells were stimulated with TNFα (100 ng mL^−1^) for the next 21 h or cell medium as a control. Cell media were collected and subjected to ELISAs. Three independent experiments were conducted.

### Test for Muco‐Adhesion of Nanocarriers to Differentiated Gingival Keratinocytes Under Static Conditions

OKG4 of 2.5 × 10^4^ cells/well were seeded onto black 96‐well plates with clear bottom (Corning #3603, Corning, NY, USA) and cultured in DermaLife K medium containing 60 µM Ca^2+^. When the cells reached 90 – 95% confluence, the differentiation of OKG4 cells was initiated by increasing the Ca^2+^ concentration to 1.4 mM for 48 h. The cell medium was then removed and replaced with sterile‐filtered saliva (20 µL) or fresh medium (20 µL). Cells were further incubated at 37 °C for 20 min. ICC‐coupled CMS nanocarriers (50 µg mL^−1^) were then added to the saliva‐coated or medium‐coated cells and incubated at 37 °C for the indicated times. To remove unbound nanocarriers, the cells were washed three times with PBS. Fluorescence of bound nanocarriers was determined using a GloMax Discover fluorescence microplate reader (Promega, Madison, WI, USA) with an excitation wavelength of 520 nm and an emission wavelength in the range of 580–640 nm. Three independent experiments were performed, each consisting of at least three technical replicates. As a loading control, the fluorescence of the input of each CMS nanocarrier was assessed. Cells incubated with medium only were used as a negative control. The negative control values were subtracted from the sample and loading control values. Using the corrected values, the amount of bound nanocarrier was calculated as follows:

(1) $$\frac{substracted\;value\;of\;the\;sample}{substracted\;value\;of\;loading\;control}\times amount\;of\;nanocarrier\;in\;loading\;control$$

### Histological Staining of 3D Oral Mucosal (Hematoxylin‐Eosin Staining)

Hematoxylin and eosin staining was performed to evaluate tissue morphology and composition. For staining, microsections were placed on slides (Superfrost Plus, Menzel GmbH & Co KG. Braunschweig, Germany) and incubated in 96% and 70% ethanol for 1 min each. The microsections were then rinsed with distilled water and incubated with Mayer's hemalum solution (Merck, Darmstadt, Germany) for 5 min. Afterwards, the microsections were rinsed with distilled water and then rinsed with tap water for 15 min. Subsequently, the microsections were incubated in distilled water for 2 min, before they were incubated with eosin solution (1:1000 with acetic acid, Merck, Darmstadt, Germany) for 5 min and then washed with distilled water. Finally, the microsections were briefly immersed in 70% and then 96% ethanol. Microscopic evaluation was performed with a light microscope (Nikon, Tokyo, Japan).

### Statistical Analysis

For statistical analysis (GraphPad Prism), the normal distribution of values was assessed with the D'Agostino‐Pearson normality test and confirmed with the Shapiro‐Wilk normality test. For normally distributed values, the unpaired Student t‐test was used to analyze the significant difference between two conditions. Otherwise, the Mann‐Whitney‐U test was used to determine the significant difference. For the ELISA assay, the mean values obtained from control cells were set at 1 as the reference for the test groups.

### Final Approval

All authors gave their final approval and agreed to be accountable for all aspects of the work.

## Conflict of Interest

All authors declare no conflict of interest.

## Supporting information



Supporting Information

## Data Availability

The data that support the findings of this study are available from the corresponding author upon reasonable request.
