# Supplementary material for: Topical Application of Dexamethasone‐Loaded Core‐Multishell Nanocarriers Against Oral Mucosal Inflammation
Source: Macromol Biosci. 2024 Oct 3;24(12):2400286. doi: 10.1002/mabi.202400286 (PMC11648588; doi:10.1002/mabi.202400286)
Supplement: Supplementary file 1 — Supporting Information [file MABI-24-2400286-s001.docx]

Supporting Information

**Topical Application of Dexamethasone-Loaded Core-Multishell Nanocarriers against Oral Mucosal Inflammation**

Cynthia V Yapto^1,2,5^, Keerthana Rajes^2^, Antonia Inselmann^1,5^, Sven Staufenbiel^3^, Kim N Stolte^1,5^, Maren Witt^4,5^, Rainer Haag^2^, Henrik Dommisch^4,5,^ *^†^*, Kerstin Danker^1,^ *^†^*

*^1^ Institute for Biochemistry, Charité - Universitätsmedizin Berlin, 10117 Berlin, Germany.*

*^2^ Freie Universität Berlin, Institute for Chemistry and Biochemistry, 14195 Berlin, Germany.*

*^3^ Freie Universität Berlin, Institute for Pharmacy, Pharmaceutical Technology, 12169 Berlin, Germany.*

*^4^ Department of Periodontology, Oral Medicine and Oral Surgery, Department of Periodontology, Oral Medicine and Oral Surgery, Charité - Universitätsmedizin Berlin, 14197 Berlin, Germany*

*^5^ Charité - Universitätsmedizin Berlin, corporate member of Freie Universität Berlin, Humboldt-Universität zu Berlin, and Berlin Institute of Health.*

*† Contributed equally*


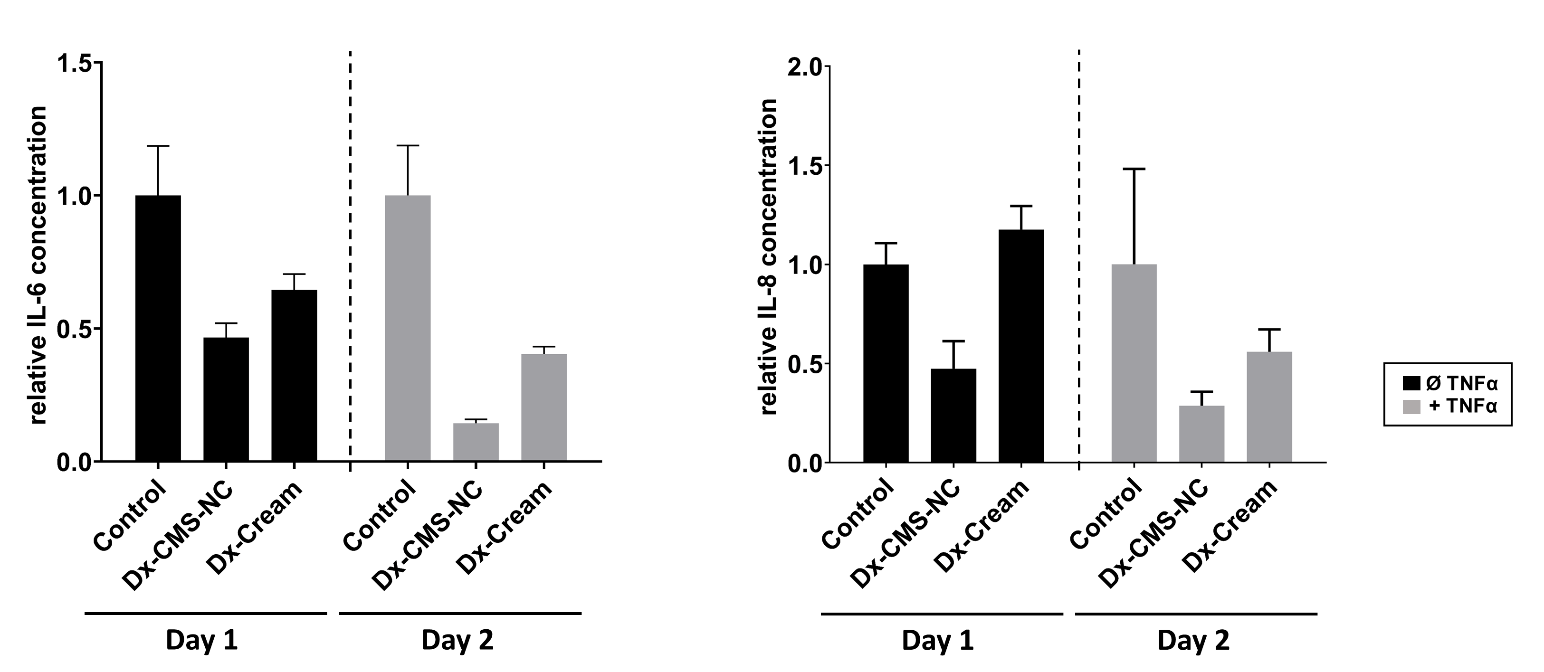


**Figure S1.** Analysis of IL-6 and IL-8 concentrations in 3D models composed of primary gingival keratinocytes and fibroblasts after treatment with Dx-CMS or Dx-cream in the absence (day 1; black bar) and presence (day 2; gray bar) of TNFα. This experiment was performed once. All ELISA experiments were performed with at least three technical replicates each. Error bar represents the standard deviation.


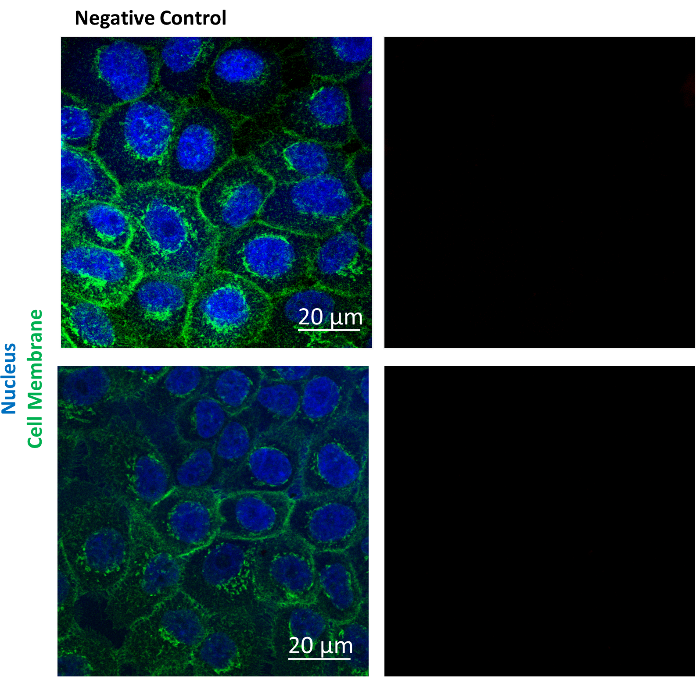


**Figure S2.** Negative controls of the dynamic assay of ICC-CMS-NC shown in Figure 5A-D. For the negative control, cells were incubated for 1 h with water. No ICC-CMS-NC signals (red) were detected in negative controls. The green colour represents the cell membrane (stained with WGA) and blue represents the cell nuclei (stained with DAPI). The images on the right show the red channel signal.

**
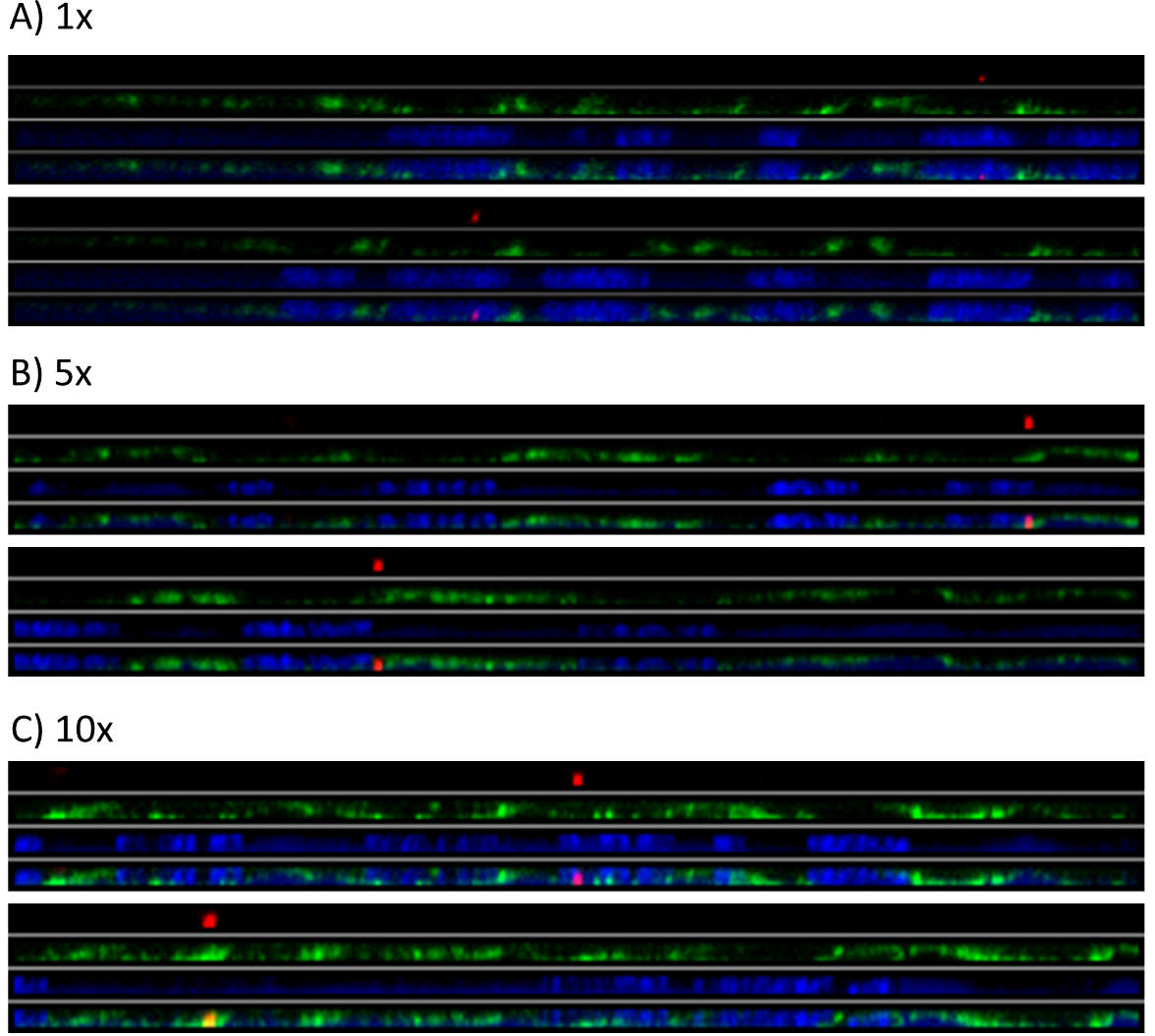
**

**Figure S3.** The orthogonal xz projections of images shown in Figure 5. For each image, two different sites were chosen to analyze the cellular localization of fluorescence-labelled CMS nanocarrier (red). The green colour represents the cell membrane (stained with WGA) and blue represents the cell nuclei (stained with DAPI).

**Figure S4.** Calibration curve for the determination of dexamethasone concentration by HPLC measurement.

**Table S1.** The cytokine array map depicts the locations of 42 cytokines, including six positions each for (Pos) and negative controls (Neg). Ang – Angiogenin, Onco M – Oncostatin M, TP - Thrombopoietin.

|  | **A** | **B** | **C** | **D** | **E** | **F** | **G** | **H** | **I** | **J** | **K** | **L** |
| --- | --- | --- | --- | --- | --- | --- | --- | --- | --- | --- | --- | --- |
| **1** | Pos | Pos | Neg | Neg | ENA-78 | GCSF | GM-CSF | GRO | GRO-α | I-309 | IL-1α | IL-1β |
| **2** | Pos | Pos | Neg | Neg | ENA-78 | GCSF | GM-CSF | GRO | GRO-α | I-309 | IL-1α | IL-1β |
| **3** | IL-2 | IL-3 | IL-4 | IL-5 | IL-6 | IL-7 | IL-8 | IL-10 | IL-12 p40/p70 | IL-13 | IL-15 | IFN-γ |
| **4** | IL-2 | IL-3 | IL-4 | IL-5 | IL-6 | IL-7 | IL-8 | IL-10 | IL-12 p40/p70 | IL-13 | IL-15 | IFN-γ |
| **5** | MCP-1 | MCP-2 | MCP-3 | MCSF | MDC | MIG | MIP-1 δ | RANTES | SCF | SDF-1 | TARC | TGF-β1 |
| **6** | MCP-1 | MCP-2 | MCP-3 | MCSF | MDC | MIG | MIP-1 δ | RANTES | SCF | SDF-1 | TARC | TGF-β1 |
| **7** | TNFa | TNF-β | EGF | IGF-1 | Ang | Onco M | TP | VEGF | PDGF BB | Leptin | Neg | Pos |
| **8** | TNFa | TNF-β | EGF | IGF-1 | Ang | Onco M | TP | VEGF | PDGF BB | Leptin | Neg | Pos |

**Table S2.** Quantitative analysis of cytokine arrays incubated with the culture medium of TNFα-treated and untreated cells revealed a TNFα-dependent upregulation of certain cytokines that just failed to reach the defined significance level of a two-fold change compared to the control.

| Cytokines | Fold Change |
| --- | --- |
| IL-8 | 1.570 |
| GRP | 1.649 |
| TNF-ß | 1.540 |
| IL-1α | 1.849 |
| MCP-1 | 1.886 |
